# The Potential Therapeutic Effect of Orexin-Treated versus Orexin-Untreated Adipose Tissue-Derived Mesenchymal Stem Cell Therapy on Insulin Resistance in Type 2 Diabetic Rats

**DOI:** 10.1155/2022/9832212

**Published:** 2022-01-17

**Authors:** Amy F. Boushra, Rania H. Mahmoud, Shymaa E. Ayoub, Rehab A. Mohammed, Hanan A. Shamardl, Amani M. El Amin Ali

**Affiliations:** ^1^Department of Medical Physiology, Faculty of Medicine, Fayoum University, Egypt; ^2^Department of Medical Biochemistry and Molecular Biology, Faculty of Medicine, Fayoum University, Egypt; ^3^Department of Medical Pharmacology Faculty of Medicine, Fayoum University, Egypt

## Abstract

Type 2 diabetes mellitus is a chronic metabolic disease characterized by resistance to peripheral insulin actions. Mesenchymal stem cells have been studied for years in T2DM therapy, including adipose tissue-derived mesenchymal stem cells (AD-MSCs). Orexin neuropeptides (A and B) are well-known regulators of appetite and physical activity. The aim of this work was to elucidate the possible therapeutic effect of AD-MSC preconditioning with orexin A (OXA) on insulin resistance in rats. Twenty-eight adult male albino rats were divided into 4 equal groups: a normal control group and 3 diabetic groups (a control T2DM group, diabetic rats treated by an AD-MSCs group, and diabetic rats treated by AD-MSCs preconditioned with OXA). We noticed that the treated groups showed a significant alleviation of insulin resistance parameters as shown in lowering the serum levels of glucose, insulin, total cholesterol, inflammatory markers, and HOMA-IR as compared to the control diabetic group with more significant reduction observed in the OXA-pretreated AD-MSCs-administrated group. More improvement was also noted in the glucose uptake and GLUT-4 gene expression in the skeletal muscle and adipose tissue in the OXA-pretreated AD-MSCs-administrated group compared to the untreated diabetic group. *Conclusion*. Preconditioning of AD-MSCs with OXA can significantly increase their potential to reduce the insulin resistance in the rat model of T2DM.

## 1. Introduction

Diabetes mellitus is a common public health problem worldwide, with extensive medical, economic, and social consequences. Type 2 diabetes (T2D) is known as the adult-onset or non-insulin-dependent diabetes (NIDDM). It accounts for over 90% of all diabetics [1]. Obesity is the most common risk factor for the development of T2D. The link between insulin resistance (IR) and T2D was confirmed for a long time. T2D is a complex metabolic disorder characterized by alterations in lipid metabolism, hyperglycemia, basal hyperinsulinemia, insulin resistance, and pancreatic *β*-cell dysfunction [2].

Inverse correlation has been recently detected between increasing body mass index and decreasing insulin receptor expression and insulin signaling in visceral adipose tissue in both humans during progressing from normal to mild and severe obesity and HFD-induced model of obesity in mice as well. The same study demonstrated that hypoxia-induced downregulation of insulin receptor expression was ameliorated by the restoration of oxygen supply. They presumed that low oxygen tension induces miR-128 upregulation and thereafter speeding up the degradation of insulin receptor mRNA [3].

Mesenchymal stem cells (MSCs) are multipotential nonhematopoietic stromal cells that have many advantages such as the ability to self-renew, low immunogenicity, and multilineage differentiation in response to chemical, hormonal, or structural stimuli. MSCs can be isolated from a variety of tissues, such as the umbilical cord, endometrial polyps, menstrual blood, bone marrow, and adipose tissue. There are likely more sources of MSCs waiting to be discovered [4]. Adipose-derived mesenchymal stem cells (AD-MSCs) are known to be differentiated into different lineages including ectodermal, mesodermal, and endodermal lineages. They have been reported to be involved in the treatment of cardiovascular and immune disorders, wound scar healing, and remodeling, radiation injury, and skin engineering [5]. Many studies reported that differentiated AD-SCs have moreover been beneficial in bone tissue engineering, liver function recovery, insulin production, demyelinating lesions, and myocardial infarction.

The orexin A (OXA, hypocretin-1) and orexin B (OXB, hypocretin-2) are multifunctional hypothalamic neuropeptides that play key roles in the regulation of feeding behavior, sleep-wakefulness rhythm, neuroendocrine homeostasis, and metabolic rate [6, 7]. Orexin A modulates the vasopressin system by increasing basal sympathetic activity and stimulating the renal and adrenal orexin receptors. Also, it has a role in regulating glucose homeostasis by interacting with pancreatic *α*- and *β*-cells [8].

This study was designed to compare the potential effect of injection of adipose tissue-derived mesenchymal stem cells preconditioned with OXA to the effect of untreated mesenchymal stem cells on the amelioration of insulin resistance in type 2 diabetic rats.

## 2. Materials and Methods

### 2.1. Animals and Experimental Design

**Figure 1:**
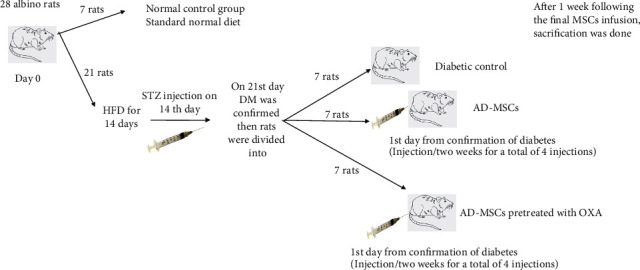
Schematic figure illustrating the timeline of the experimental protocol.

Animals were purchased from the animal care unit of the Faculty of Medicine, Cairo University. All ethical rules for animal management were followed. All experimental procedures were approved by the Institutional Animal Care Committee of Cairo University Animal Care Center (R.205), Cairo, Egypt. Twenty-eight adult male albino rats were weighing 150-200 gm. Animals housed 7 per cage in a constant temperature (22-25°C) with a 12 h alternating light-dark cycles and had free access to food and water. Animals divided randomly into four study groups, each containing 7 rats. The schematic figure illustrating the timeline of the experimental protocol is shown in Figure 1: group I, normal control group; group II, diabetic control T2DM rats with no treatment given; group III, T2DM rats received four infusions of AD-MSCs (one infusion every two weeks); and group IV, T2DM rats received AD-MSCs pretreated with OXA (for a total of four infusions, one infusion/two weeks).

### 2.2. Induction of Type 2 Diabetes

Beginning on day 0, animals were divided into two groups: the normal control group (*n* = 7) was fed a standard normal diet and the diabetic groups (*n* = 21) were fed a high-fat diet (HFD; 20% protein, 60% fat, and 20% carbohydrates) for a period of two weeks. On day 14, rats on HFD were injected intraperitoneally with a single low dose of streptozotocin (STZ, 45 mg/kg) to induce T2DM [9]. Both the low doses of STZ and HFD are essential elements to induce T2DM with insulin resistance [10]. Subsequently, all rats had free access to food and water and were continued on their respective diets till the end of the study. On day 21, fasting blood glucose (FBG) and insulin levels were measured in each of the control and diabetic groups after an overnight fast. Additionally, oral glucose tolerance tests (OGTTs) were measured to confirm T2DM in HFD associated with STZ group. Insulin resistance was estimated using insulin resistance index (HOMA-IR). The established T2DM model rats were divided in to 3 groups: diabetic control (T2DM group), AD-MSCs-treated group, and AD-MSCs pretreated with OXA-administrated group (*n* = 7 each). AD-MSCs and AD-MSCs preconditioning with OXA-treated groups were infused with 2 × 10^6^ MSCs suspended in 0.3 ml physiological saline through the tail vein every two weeks, for a total of four infusions [11]. Rats in the normal control group and the control diabetic group were administered only 0.3 ml physiological saline through the tail vein every two weeks for a total of four infusions as in the other two groups.

### 2.3. Adipose-Derived MSC Isolation, Culture, and Identification

Adipose-derived MSCs were isolated and prepared from adipose tissue taken from the inguinal regions and flanks of immature rats [9, 12]. Adipose tissue was digested by collagenase type II (Sigma, Germany) and dissolved in Phosphate Buffer Saline (PBS; Biodiagnostic Co., Giza, Egypt) for 2 hrs at 37°C. Following filtration to eliminate all tissue debris, centrifugation was done at 600 × *g* for 10 min at room temperature to form a cell pellet. Cell pellet was cultured in an RPMI medium (Gibco; Thermo Fisher Scientific, Inc., Waltham, MA, USA) and supplemented with 10% fetal bovine serum (Gibco; Thermo Fisher Scientific, Inc.), penicillin (80 U/ml), and streptomycin (0.2 mg/ml) at 37°C in a cell culture incubator containing 5% CO_2_ and a relative humidity of ~100%. AD-MSCs were identified in culture by their characteristic morphology, spindle-shaped cells. MSC phenotypes of the third passage (P3) were analyzed by flow cytometry. Once 80-90% adipose MSC confluence was reached, cells were separated using 0.25% trypsin-EDTA (Gibco; Thermo Fisher Scientific, Inc.) and resuspended in other flasks. Cells were then washed with PBS and incubated for 15 min at room temperature in the dark with antibodies for CD29, CD34, and CD90 surface markers for additional identification and characterization of AD-MSCs using flow cytometry analysis (Beckman Coulter). The fourth passage (P4) of AD-MSCs was used in the whole experiment.

### 2.4. Labeling of BM-MSCs with PKH26

MSCs were labeled with PKH26 (Sigma-Aldrich, Germany). Cells were first centrifuged, washed twice in serum-free medium and were pelleted and suspended in dye solution. Cells were injected intravenously into rat tail vain. At the end of the study, muscle and adipose tissues were examined with a fluorescence microscope to detect and trace the cells [13].

### 2.5. Preconditioning of AD-MSCs with Orexin A

The MTT assay was achieved for preconditioning of AD-MSCs with orexin A as follow: AD-MSCs were cultured in 96-well plates (2 × 10^6^ cells/ml) with 200 *μ*l culture medium (DMEM/F12; Gibco, Thermo Fisher Scientific, Inc.) with OXA at concentration of 100 nM. 20 *μ*l MTT solution (5 mg/ml) was added to each well and incubated for 4 h. The medium was then aspirated and 200 *μ*l dimethyl sulfoxide was added. The absorbance value was measured using a multi-well spectrophotometer (Bio-Rad, USA) at 490 nm [14]. OXA was obtained from Sigma Aldrich (Darmstadt, Germany).

### 2.6. Determination of the Effects of Infused MSCs on Hyperglycemia and Insulin Resistance in T2D Rats

Following each MSCs injection, fasting blood glucose levels were detected with a glucometer (Accu-Chek Meter; Roche Diagnostics GmbH, Germany). At one week following the administration of the final MSCs infusion, OGTTs were performed again to rats of different groups. Blood samples were obtained from rats by squeezing the caudal vein. Following this, serum was isolated from whole blood samples via centrifugation at 800 x g for 10 min at 4°C. The fasting serum insulin (FINS) levels were measured using an ELISA assay kit (Sunglong Biotech, Hangzhou, China) 1 week following the final injection of MSCs. In addition, the homeostatic model assessment of insulin resistance indexes (HOMA-IR) was used to assess changes in insulin resistance calculated according to the following equation: HOMA − IR = [fasting blood glucose (mmol/l) × fasting serum insulin (mU/l)/22.5 [9].

### 2.7. Oral Glucose Tolerance Test (OGTT)

Oral glucose load of 2 gm per kg body weight was given with an oral gavage following overnight fasting (12 h). The blood samples were collected from the tail vein at time 0 (prior to glucose load), 30, 60, 90, and 120 min after the glucose load for measurement of blood glucose levels [15].

### 2.8. Sacrification and Chemistry

At the end of experiment, the rats were anesthetized with intraperitoneal pentobarbital sodium (60 mg/kg) and sacrificed by cervical dislocation [16]. The soleus muscles were dissected, one used to assess insulin-dependent glucose uptake. The soleus muscle of the other side was used to assess glucose transporter-4 (GLUT-4) gene expression by inserting in liquid nitrogen and stored at -80°C until analysis.

### 2.9. Biochemistry

Total cholesterol (TC), triglyceride (TG) and HDL-cholesterol (HDL-C) were assayed in serum samples by colorimetric determination method using commercially available kits obtained from Biodiagnostic, Egypt. Interleukin-6 (IL-6) and tumor necrosis factor-*α* (TNF-*α*) serum levels were analyzed by ELISA kits according to the manufacturer's instructions (Cusabio Biotech, China).

### 2.10. Quantitative Real Time PCR Analysis

Total RNA from soleus muscle and adipose tissues homogenates was extracted using tissue extraction kit (RNeasy Mini Kit, Qiagen, Germany) according to the manufacturer's instructions. The concentration of the obtained RNA and the purity were checked using UV spectrophotometer. RNAs were reverse transcribed to cDNA using the quantiscript cDNA synthesis kit (Qiagen, Germany). The cDNA was amplified with QuantiTect SYBR® Green PCR Kits (Qiagen, Germany) to form 25-*μ*l reaction volume. RT-PCR reactions were carried out using TaqMan® gene expression assays for GLUT 4 and AKT2 genes. The gene-specific primer sequences were as follow: forward; 5′-ACA ATG TCT TGG CTG TGC TG-3′ and reverse; 5′-TCC CAC ATA CAT AGG CAC CA-3′ for GLUT 4, forward; 5′-TAT ACC GCG ACA TCA AGC TG-3′ and reverse; 5′- GGT CCC ACA GAA GGT TTT CA -3′ for AKT2 and forward; 5′- CAC CCT GTT GCT GTA GCC ATA TTC-3′ and reverse 5′- GAC ATC AAG AAG GTG GTG AAG CAG-3′ for GAPDH. Amplification conditions were as follows: 15 min at 95°C for enzyme activation, then 40 cycles of 15 seconds at 94°C for denaturation, 30 seconds at 60°C and finally 30 seconds at 72°C for annealing and extension. The expression mRNA levels of GLUT4 and AKT2 genes were normalized to the glyceraldehyde-3-phosphate dehydrogenase (GAPDH) as internal control gene using the 2-^*ΔΔ*Ct^ method [17]. the relative value for the control group expression levels was set as one.

### 2.11. Glucose Uptake Measurement in Isolated Soleus Muscles

After isolation of soleus muscles of rats, glucose transport was measured by the use of glucose analog 2-[^3^H]DG. Muscle strips were incubated in a shaking water bath at 30°C for 30 min into 25-ml flasks, each is containing 3.0 ml of oxygenated Krebs-Ringer bicarbonate (KRB) buffer supplemented with 8 mM glucose, 32 mM mannitol, and 0.1% BSA [radioimmunoassay (RIA) grade]. Flasks were gassed continuously with 95% O2-5% CO2 throughout the experiment. One flask containing only 3 ml of incubation medium with no added tissues was used as control.

Insulin (soluble porcine) was added (12 nM). At this concentration, insulin activates glucose transport maximally in soleus muscles. Muscles were next washed for 10 min at 29°C in 3 ml of KRB buffer containing 40 mM mannitol and 0.1% BSA. Muscles were then incubated for 20 min at 29°C in 3 ml of KRB buffer containing 8 mM 2-[^3^H] DG, 32 mM [^14^C] mannitol, 2 mM sodium pyruvate and 0.1% BSA. Insulin was present throughout the wash and uptake incubations. After incubation, the muscle was immediately dried on filter paper and then weighed. The 2-[^3^H] DG level was determined in 1 ml of each sample as well as in 1 ml of the control. The insulin-stimulated glucose uptake by the muscle was calculated in mg/g tissue/h of incubation. This concentration of insulin is considerably greater than that required to stimulate maximum glucose uptake in this preparation. Glucose uptake = (Glucose conc. of control sample-sample glucose conc. After 1 h) ×3(medium volume)/(100 × weight of muscle in g). 2-[^3^H] DG uptake rates were corrected for extracellular trapping using [^14^C] mannitol [18, 19].

### 2.12. Glucose Uptake Measurement in Adipose Tissue

Glucose transport in isolated adipocytes was analyzed by the use of radiolabelled 2-deoxyglucose. Subcutaneous fat pads were excised from rats and digested by collagenase (1 mg/ml) at 37°C for 30 min with constant shaking (100 rpm). Digestion was performed using glucose and insulin free Krebs-Ringer bicarbonate (KRB) buffer supplemented with 30 mM HEPES, 2.5% bovine serum albumin and 200 nM adenosine. Following, isolated adipocytes treated with insulin (100 nM) for 30 minutes. 2-deoxy-D glucose (3 *μ*M) was added for 60 minutes. The reaction was terminated and glucose uptake was stopped by spinning the suspension through dinonyl phthalate oil [20].

## 3. Statistical Methods

Statistical analysis was performed using the arithmetic mean, standard deviation (S.D.) after checking of normality of distribution, analysis of variance (one-way ANOVA) and comparison between each two groups using post Hoc Tukey test, using the statistical SPSS software for Windows, Version 18 (SPSS Inc., Chicago, USA). For interpretation of results of tests of significance, significance was detected at P< 0.05.

## 4. Results

### 4.1. Identification and Characterization (Immunophenotypes of AD-MSCs)

**Figure 2:**
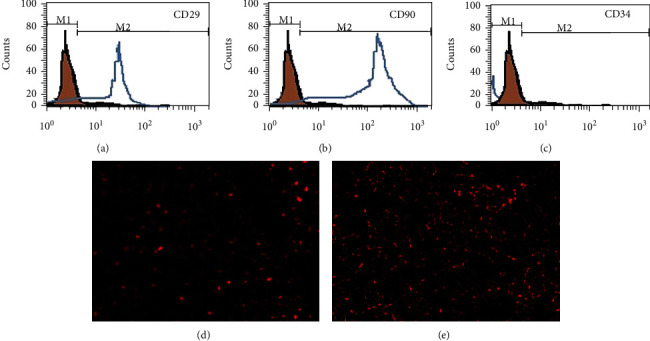
Characteristics and identification of AD-MSCs. The MSCs were identified by their immunological phenotypes; the isolated and cultured cells were positive for CD29 (a) and CD90 (b) and were negative for CD34 (c). Muscle (d) and adipose tissue (e) sections were investigated by fluorescence microscope which revealed homing of AD-MSCs labeled with PKH26 fluorescent dye (red fluorescence).

It was examined by flow cytometry. AD-MSCs cells were negative for the hematopoietic marker (CD34), while strongly positive for mesenchymal stem cell-specific markers including CD29 and CD90. MSCs infusion alleviates hyperglycemia in T2D rats. Infused MSCs were identified in advance by their phenotypes (Figures 2(a)–2(c)).

### 4.2. Homing of Labelled AD-MSCs

Muscle and adipose tissue sections were investigated by fluorescence microscope which revealed homing of AD-MSCs labeled with PKH26 fluorescent dye (red fluorescence) (Figures 2(d) and 2(e)).

### 4.3. Level of Fasting Glucose, Insulin Levels, and HOMA-IR in the Studied Groups

As regards the level of fasting glucose, insulin and HOMA-IR profiles after induction of diabetes and before treatment there was no significant differences between the three diabetic groups (diabetic control group, AD-MSCs treated group and group administrated AD-MSCs pretreated with OXA). In addition, a significant increase in fasting glucose, serum insulin levels and HOMA-IR was detected when we compared each of the three diabetic groups (before treatment) with normal control group (*p* < 0.0001) (Table 1).

As shown in Table 2, control diabetic rats (group II, not received any treatment) exhibited a significant increase in fasting glucose, serum insulin levels, and HOMA-IR compared to that of the normal control group (group I) (*p* < 0.001). However, it was found that treatment with AD-MSCs in diabetic group treated with stem cells (group III) produced a significant decrease in the mean ± SD values of fasting glucose, serum insulin, and HOMA-IR when compared to diabetic group (*p* < 0.001). Moreover, treatment with AD-MSCs preconditioned with OXA (group IV) produced more significant reduction in all values compared to groups II and III (*p* < 0.001 each). This means that the OXA treated stem cells show better results than stem cells untreated with OXA. There was no significant difference (*p* > 0.05) between group I and group IV regarding serum glucose and HOMA-IR levels. These results demonstrate that stem cell preconditioned with OXA has the best blood-glucose-lowering effect and improves the insulin resistance in STZ induced diabetes in rats.

### 4.4. Oral Glucose Tolerance Curve

**Figure 3:**
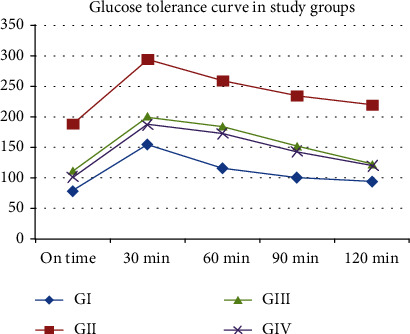
Glucose tolerance curve in the study groups. GI: control group; GII: diabetic group; GIII: diabetic group treated with stem cells; GIV: diabetic group treated with stem cells preconditioned with orexin A.

The curve of the oral glucose tolerance test shows that all groups had an increase in the blood glucose levels upon administration of the glucose load as detected at 30 min. This high level further decreased significantly with time to approach fasting levels in diabetic treated rats (groups III and IV), compared to control diabetic rats in group II which remained persistently high (*p* < 0.05). The lower glucose level was found among group IV and that confirms the lowering effect of stem cell preconditioned with OXA in control of diabetes (Figure 3).

### 4.5. Levels of Lipid Profile and Inflammatory Markers in the Studied Groups

Table 3 illustrates that there is a statistically significant difference with *p* value < 0.05 between different study groups as regards lipid profile. Control diabetic rats (group II) showed a statistically significant increase in serum triglycerides (TG) and total cholesterol (TC) with a significant decrease in HDL levels compared to other groups. While significant decrease in both TG and TC and increase in HDL levels were observed by treatment with AD-MSCs in group III compared to group II. These values exhibited more significant improvement after treatment with AD-MSCs preconditioned with OXA compared to group II and group III.

In control diabetic rats, TNF-*α* and IL-6 significantly increased compared to other groups. Treatment with AD-MSCs and AD-MSCs preconditioned with OXA were reported to decrease TNF-*α* and IL-6 levels significantly as compared to group II which can improve insulin resistance (*p* < 0.001). So, treatment with stem cells can improve the lipid profile and inflammatory condition but orexin treated stem cells show better results than stem cells alone.

### 4.6. Levels of Glucose Uptake in Skeletal Muscle and Adipose Tissue in the Studied Groups

To prove the hypoglycemic effect at the cellular level involved with administration of AD-MSCs and AD-MSCs preconditioned with OXA on the improvement of insulin resistance, we assessed the glucose uptake in skeletal muscle and adipose tissue. In the control diabetic rats (group II), glucose uptake in skeletal muscle and adipose tissues was significantly decreased compared to normal control group (*p* < 0.001). However, glucose uptake was significantly (*p* < 0.001) increased by treatment with AD-MSCs in group III compared to group II and more significant improvement noted after treatment with AD-MSCs preconditioned with OXA in group IV compared to each of groups II and III (*p* < 0.001 each). Thus, these results suggest that AD-MSCs preconditioned with OXA improve the sensitivity of target tissues to insulin action (Table 3).

### 4.7. Expression Levels of GLUT-4 and AKT2 in Skeletal Muscle and Adipose Tissue in the Studied Groups

**Figure 4:**
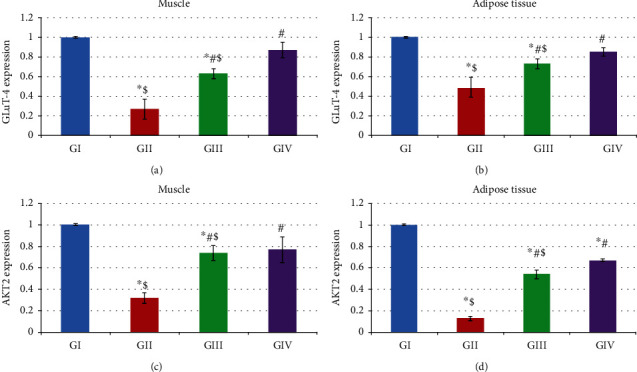
The effect of administration of AD-MSCs and AD-MSCs pretreated with OXA on the gene expression levels of GLUT-4 in the (a) muscle and (b) adipose tissue and AKT2 in the (c) muscle and (d) adipose tissue of T2DM rats. Results are expressed as mean ± SD, (*n* = 7). ∗Significant as compared to the control group (GI). ^#^Significant as compared to the T2DM group (GII). ^$^Significant as compared to AD-MSCs preconditioned with the OXA group (GIV). GI: normal control group; GII: diabetic group; GIII: diabetic group treated with stem cells; GIV: diabetic group treated with stem cells preconditioned with OXA. *p* < 0.05 is adopted as a significant value.

To determine the possible underlying mechanism involved with AD-MSCs and AD-MSCs preconditioned with OXA on the improvement of insulin resistance, we assessed GLUT-4 expression in skeletal muscle and adipose tissue. GLUT-4 expression in skeletal muscle and adipose tissues significantly decreased in control diabetic rats compared to the normal control group (*p* < 0.05) and it was significantly increased by treatment with AD-MSCs in group III compared to group II. More significant improvement was noted after treatment with AD-MSCs preconditioned with OXA in group IV compared to each of groups II and III (*p* < 0.05). Thus, these results suggest that AD-MSCs preconditioned with OXA might mediate GLUT-4 expression and accordingly improve the sensitivity of target tissues to insulin action (Figure 4). To determine whether impaired GLUT-4 was linked to changes in the AKT2 or not, we measured it in both skeletal muscle and adipose tissues. We found that AKT2 mRNA gene expression was significantly decreased in both muscle and adipose tissue in the control diabetic rats compared to the normal control group (*p* < 0.05). Treatment with AD-MSCs in group III significantly increases AKT2 gene expression in both skeletal and adipose tissue when compared to group II (*p* < 0.05) and these values were significantly more upregulated after treatment with AD-MSCs preconditioned with OXA in (group IV), when compared to group II (*p* < 0.05) (Figure 4).

## 5. Discussion

Type 2 diabetes is a global health burden affecting millions worldwide. It is a metabolic disorder associated with chronic hyperglycemia due to insulin resistance and deteriorated *β*-cell function and consequently decreased insulin secretion [21]. Many treatment approaches have been tried but the target treatment goals have not been achieved yet, besides the side effects of the long-term therapies. Stem cell therapy is an emerging area of research for diabetes. Sources for stem cell therapies in DM are multiple, including embryonic stem cells, cord blood stem cells and mesenchymal stem cells which have shown their benefits in the long-term treatment of diabetes [22]. AD-MSCs attracted more attention, among the other sources of mesenchymal stem cells, as a recent therapeutic approach for T2DM. Many factors were considered concerning the applicability of AD-MSCs including the easy, less invasive obtainment and the convenient harvest [23].

We hypothesized that infused AD-MSCs might contribute to amelioration of the insulin resistance of peripheral insulin target tissues and AD-MSCs treated with OXA can give more better effects. To test this hypothesis, we induced a diabetic rat model then evaluated the therapeutic effects of AD-MSCs infusion and explored the possible effect of pretreatment of AD-MSCs with OXA.

Our results proved the occurrence of hyperglycemia, hyperinsulinemia, increased insulin resistance as indicated by HOMA-IR and impaired glucose tolerance curve in the diabetic untreated group compared to normal control group. Following AD-MSCs therapy, the diabetic rats showed an improvement in the level of fasting glucose, insulin levels and HOMA-IR compared to untreated diabetic rats. While, the rats treated with AD-MSCs preconditioned with OXA produced a more significant reduction in all values compared to rats treated with AD-MSCs only. This means that the OXA treated stem cells show better results than stem cells untreated with OXA. Furthermore, the least glucose levels were found among rats treated with AD-MSCs preconditioned with OXA in the curve of oral glucose tolerance test which confirms the lowering effect of stem cell preconditioned with OXA in control of diabetes. The previous results are in agreement with Zihui et al. who infused MSCs 4 times, once/2 weeks for 8 weeks. Their results demonstrated improved hyperglycemia and insulin resistance in diabetic rats [11]. Moreover, the treatment with human AD-MSCs suspension reduced area under the curve (AUC) indicating better glucose tolerance [24].

Our results showed that infused AD-MSCs reduced blood glucose levels in diabetic rats and improving insulin sensitivity possibly by significant upregulation of GLUT4 and Akt2 gene expression levels in muscle and adipose tissues. In addition, diabetic rats received AD-MSCs treated with OXA showed more significant improvement which reveal better insulin signaling and improvement of the sensitivity of target tissues to insulin action.

Furthermore, obvious amelioration in dyslipidemia (lower triglycerides, total cholesterol and higher HDL) and decreased inflammatory markers (TNF-*α* and IL6) levels have been noticed after administration of AD-MSCs treated with OXA.

In 2017, Shree and Bhonde established 2 insulin resistant cell models and treated them with AD-MSCs conditioned media. They found that the conditioned media from adipose derived mesenchymal stem cells treated cells stimulated glucose uptake, showed inhibition of adipogenesis and significant reduction of intramuscular triglyceride accumulation that contribute to insulin resistance [2]. A drastic upregulation of GLUT4 gene and significant reduction in IL6 gene was also observed, indicating possible mechanism of glucose uptake and increased insulin sensitivity associated with a decrease in inflammation. We also found an increase in GLUT4 and Akt2 protein expression that might lead to improved glucose uptake and enhanced insulin signaling, respectively.

In 2016, Wojciechowicz1 et al. studied the effects of 24 h exposure of OXA and OXB in the culture medium, on porcine preadipocyte proliferation and differentiation [25]. They found that both OXA and OXB in high doses could increase the preadipocyte proliferation as well as their differentiation into mature adipocytes, so both peptides stimulate white adipogenesis. Wei et al. also concluded that treatment with OXA but not OXB inhibited bone marrow mesenchymal stem cells differentiation into osteoblast and increased their differentiation into adipocytes favoring bone marrow adipogenesis [26]. Moreover, it has been previously proved that stimuli of white adipogenesis can ameliorate both lipid homeostasis and insulin sensitivity in rodents [27]. That is why it is now accepted that improved insulin sensitivity due to orexin treatment in rodents may result from augmented preadipocyte proliferation and differentiation.

Zhao et al. showed that treatment with OXA enhanced neuronal proliferation and differentiation of newborn cells into neurons promoting neurogenesis in the dentate gyrus of the hippocampus [28]. Suo et al. concluded that in their study on rat hepatocytes in vitro, OX1 receptor mRNA expression and activation were upregulated by exogenous OXA in a dose-dependent manner. They suggested that OXA could increase cell proliferation and protect cells from apoptosis. They also reported that inflammatory mechanisms play a key role in the pathogenesis of DM [29]. Both TNF-*α* and IL-6 have been suggested to be associated with insulin resistance in T2DM subjects. Increased TNF-*α* expression has been observed in adipose tissue of obese rodents and human subjects contributing to obesity-associated insulin resistance and hence the development of T2DM. TNF-*α* has been suggested to negatively regulate the important insulin-sensitizing nuclear receptor PPAR*γ* (peroxisome proliferator-activated receptor gamma). TNF-*α* also has direct effects on insulin signaling plus the stimulation of lipolysis leading to elevated free fatty acids [30].

In addition, it was reported that IL-6 could reduce the expression of insulin-sensitizing adipokine (adiponectin), GLUT4, both insulin receptor substrate-1 mRNA and protein as well as tyrosine phosphorylation in 3T3-L1 adipocytes [30].

Burhans et al. observed a significant positive correlation between IL-6 and each of fasting serum insulin and HOMA-IR [31]. It was previously also reported that IL-6 gene polymorphism had lower IL-6 levels, lower area under the glucose curve after an oral glucose tolerance test, lower fasting serum insulin levels and an increased insulin sensitivity index as compared with carriers of the normal IL-6 allele, despite similar age and BMI [32].

Duffy et al. evaluated the effect of pretreatment with OXA in vitro in murine microglial and hypothalamic neuronal cell lines challenged with palmitic acid (PA) as a saturated fatty acid. They demonstrated that pretreatment with OXA reduced the pro-inflammatory markers IL-6, TNF-*α* and inducible nitric oxide synthase in microglial cells after their increase following PA exposure. Moreover, the anti-inflammatory marker arginase-1 is increased by OXA. So, they concluded that OXA has an immunomodulatory role in microglia, reducing pro-inflammatory cytokines and increasing anti-inflammatory factors to provide a favorable neuronal microenvironment [33].

Oxidative stress is reported to be one of the threats to the cultured cells. Cell culture causes oxidative stress due to more ROS generation and impairment of cellular antioxidant defenses. The elevated level of ROS leads to decreased cell proliferation, or even death. Cell culture media are frequently deficient in antioxidants [34]. Butterick et al. demonstrated that OXA decreases hydrogen peroxide-induced lipid peroxidative stress, and decreases caspase induced apoptosis in immortalized primary embryonic rat hypothalamic cell line in vitro so increases cell viability and provides neuroprotection [35].

The treatment of diabetic rats by AD-MSCs pretreated with OXA improved the biochemical parameters towards the normal suggesting the synergistic effect of the combined therapy. Nevertheless, the wide application needs more investigations to be assured.

One of the limitations of our study is that a detailed investigation of underlying mechanisms explaining the effects of orexin on AD-MSCs is missing.

## Figures and Tables

**Table 1:** Levels of fasting glucose, insulin, and HOMA-IR after induction of diabetes and before treatment.

Variables	Control group	T2DM group	AD-MSCs group	AD-MSCs pretreated with OXA group
Fasting glucose (mmol/l)	6.1 ± 0.16	17.5 ± 3.1^a^	17.4 ± 0.44^a^	17.7 ± 0.34^a^
Insulin (mU/l)	8.1 ± 0.10	18.4 ± 1.1^a^	17.3 ± 0.28^a^	17.6 ± 0.38^a^
HOMA-IR	2.2 ± 0.11	14.3 ± 2.7^a^	13.5 ± 0.43^a^	13.2 ± 0.40^a^

The results are reported as mean ± SD; *n* = 7 each. *p*^a^ < 0.0001 as compared to the control group. T2DM: type 2 diabetes; AD-MSCs: adipose-derived mesenchymal stem cells; OXA: orexin A; HOMA-IR: homeostatic model assessment of insulin resistance index. The significance level was set at *p* < 0.05.

**Table 2:** Fasting glucose, insulin, and HOMA-IR profiles in different study groups.

Variables	Control group	T2DM group	AD-MSCs group	AD-MSCs pretreated with OXA group
Fasting glucose (mmol/l)	6.1 ± 0.16	17.5 ± 3.1^a^	9.6 ± 1.2^abc^	7.1 ± 0.64^b^
Insulin (mU/l)	8.1 ± 0.10	18.4 ± 1.1^a^	11.8 ± 1.2^abc^	9.7 ± 0.46^ab^
HOMA-IR	2.2 ± 0.11	14.3 ± 2.7^a^	5.1 ± 1.2^abc^	3.1 ± 0.44^b^

The results are reported as mean ± SD; *n* = 7 each. *p*^a^ < 0.001 as compared to the control group, *p*^b^ < 0.001 as compared to the T2DM group, and *p*^c^ < 0.001 as compared to AD-MSCs pretreated with the OXA group. T2DM group/T2DM: type 2 diabetes; AD-MSCs: adipose-derived mesenchymal stem cells; OXA: orexin A; HOMA-IR: homeostatic model assessment of insulin resistance index. The significance level was set at *p* < 0.05.

**Table 3:** Levels of glucose uptake, lipid profile, and inflammatory markers in different study groups.

Variables	Control group	T2DM group	AD-MSCs group	AD-MSCs pretreated with OXA group
Glucose uptake				
Muscle (mg/g tissue/h)	8.57 ± 0.22	4.53 ± 0.19^a^	6.5 ± 0.16^abc^	8.31 ± 0.132^b^
Adipose tissue (mg/g tissue/h)	9.67 ± 0.182	3.73 ± 0.182^a^	7.64 ± 0.214^abc^	9.34 ± 0.25^b^
Lipid profile				
Triglycerides (mg/dl)	75.5 ± 2.6	118.9 ± 6.9^a^	92 ± 7.8^abc^	82.4 ± 6.2^b^
Total cholesterol (mg/dl)	136.5 ± 0.524	259.7 ± 1.07^a^	185.41 ± 0.917^abc^	137.56 ± 0.725^b^
HDL (mg/dl)	59.75 ± 0.717	27.85 ± 0.723^a^	41.11 ± 0.56^abc^	58.75 ± 0.55^b^
Inflammatory markers				
TNF-*α* (pg/ml)	15.16 ± 0.35	92.2 ± 0.74^a^	52.58 ± 0.312^abc^	15.8 ± 0.419^b^
IL-6 (pg/ml)	30.73 ± 0.319	115.21 ± 0.69^a^	68.25 ± 0.725^abc^	31.58 ± 0.453^b^

The results are reported as mean ± SD; *n* = 7 each. *p*^a^ < 0.001 as compared to the control group, *p*^b^ < 0.001 as compared to the T2DM group, and *p*^c^ < 0.001 as compared to AD-MSCs pretreated with the OXA group. T2DM group/T2DM: type 2 diabetes; AD-MSCs: adipose-derived mesenchymal stem cells; OXA: orexin A. The significance level was set at *p* < 0.05.

## Data Availability

All data are available within the manuscript.
